# Recombinant Nontypeable Genotype II Human Noroviruses in the Americas

**DOI:** 10.3201/eid2601.190626

**Published:** 2020-01

**Authors:** Kentaro Tohma, Cara J. Lepore, Juan I. Degiuseppe, Juan A. Stupka, Mayuko Saito, Holger Mayta, Mirko Zimic, Lauren A. Ford-Siltz, Robert H. Gilman, Gabriel I. Parra

**Affiliations:** US Food and Drug Administration, Silver Spring, Maryland, USA (K. Tohma, C.J. Lepore, L.A. Ford-Siltz, G.I. Parra);; INEI-ANLIS “Dr. Carlos G. Malbrán,” Buenos Aires, Argentina (J.I. Degiuseppe, J.A. Stupka);; Tohoku University Graduate School of Medicine, Sendai, Japan (M. Saito);; Universidad Peruana Cayetano Heredia, Lima, Peru (H. Mayta, M. Zimic);; Johns Hopkins University Bloomberg School of Public Health, Baltimore, Maryland, USA (R.H. Gilman)

**Keywords:** gastroenteritis, diarrhea, calicivirus, norovirus, viruses, Americas, enteric infections

## Abstract

We report multiple nontypeable genotype II noroviruses circulating in South America; nucleotides differed by >25% from those of other genotypes. These viruses have been circulating in the Americas for ≈20 years and show recombination with other genotypes. Clues to norovirus natural history can guide development of treatment and prevention plans.

Norovirus is a leading cause of acute gastroenteritis (*1*). The norovirus RNA genome is organized into 3 open reading frames (ORFs). ORF1 encodes for 6 nonstructural proteins, including the RNA-dependent RNA polymerase (RdRp). ORF2 and ORF3 encode for the major capsid protein (VP1) and minor capsid protein (VP2). Norovirus classification was recently updated, and these viruses are now classified into 10 genogroups (GI–GX) and ≈40 genotypes (*2*). This classification is based on the genetic diversity presented by VP1 and RdRp (*3*). Human noroviruses are mostly represented by the GI and GII strains.

Advances in genome sequencing approaches enabled us to detect a novel (nontypeable) GII norovirus circulating in Peru in 2008 (*4*). As part of a larger study to sequence the genomes from noroviruses circulating on different continents and over different decades, we found 7 additional nontypeable GII norovirus strains: 2 in fecal samples (PNV024019 and PNV027026) from children in Peru with diarrhea (*5*) and 5 in fecal samples collected in Buenos Aires, Argentina (*6*) (Appendix 1 Table). These strains were detected by routine PCR screening and were either incorrectly assigned as GII.22 or could not be assigned to any genotypes in the Noronet Typing Tool (*5*,*6*). To investigate the genetic structures and their evolutionary relationship with other norovirus strains, we performed norovirus GII-specific amplicon-based next-generation sequencing as described previously (*4*). We obtained nearly complete genomes for all 7 strains; average depth coverage (sequenced nucleotides/genome position) was >5,253× (GenBank accession nos. MK733201–MK733207).

The maximum-likelihood phylogenetic tree of the RdRp-encoding nucleotide sequences showed that the newly obtained strains from Argentina and Peru were closely related to each other and clustered with a group of nontypeable strains (recently classified as GII.P23–GII.P27) (*2*) (Figure). This genetic group diverged from GII.P22, GII.P25, GII.P38 (previously known as GII.Pn), and GII.P40 (previously known as GII.P22). One of the strains, Arg1382, was classified as GII.P26 under the updated classification system; however, this strain did not distinctly cluster with other GIIP26 strains on the tree. The other strains remain unclassified. 

**Figure:**
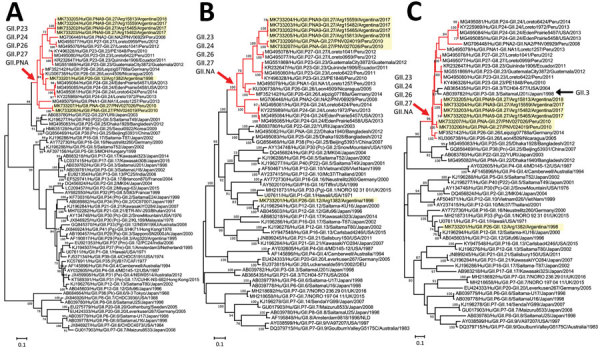
Phylogenetic analyses of nontypeable norovirus GII strains locally distributed in the Americas. Maximum-likelihood phylogenetic trees of the RNA-dependent RNA polymerase–-encoding nucleotide sequences (>771 nt) (A), major capsid protein–encoding nucleotide sequences (>1,605 nt) (B), and the minor capsid protein–encoding nucleotide sequences (>536 nt) (C) from human GII norovirus strains were created by using the Tamura-Nei model. Yellow highlighting indicates strains detected in this study; red branches and arrows indicate the divergence of a group of nontypeable GII strains reported in the Americas; and black arrow indicates GII.3 strains that diverged in the same branches with nontypeable GII strains. Bootstrap values (>70) from 100 replicates are shown on the nodes. GenBank accession numbers are shown. Scale bars indicate genetic distance (nucleotide substitutions/site).

Similar to the RdRp phylogenetic tree, the analysis of the VP1-encoding nucleotide sequences showed a distinct group of nontypeable strains (recently classified as GII.23–GII.27) (Figure). This genetic group branched out from the GII.22 and GII.25 strains (mean nucleotide difference in VP1 25.9% and mean amino acid difference 19.2%) and further diverged into different genotypes. All strains we report were assigned as GII.27 except Arg1382. Of note, Arg1382, which was the oldest virus among the strains with a nontypeable RdRp, displayed VP1 from the GII.12 genotype. 

The analysis of the VP2-encoding nucleotide sequences also showed a distinct group of those GII.23–27 genotypes; however, clustering of genotypes (particularly GII.27) seen in VP1 was disrupted in the VP2 tree (Figure). These genotypes were clustered with the VP2 sequences from GII.3 strains, suggesting possible recombination of the VP1 and VP2 among those strains. Although recombination at the ORF2/ORF3 junction region has been reported for different GII.4 variants (*7*), we showed possible recombination between different genotypes. Simplot analyses provided further support that these nontypeable strains have recombined with multiple genotypes at the ORF1/ORF2 and ORF2/ORF3 junction regions (Appendix 1 Figure 1) during their evolutionary history.

All strains in this group were detected in the Americas (United States, Nicaragua, Guatemala, Ecuador, Peru, and Argentina) (Appendix 1 Figure 2). One previous study reported strains associated with this group outside the Americas; an immunocompromised patient in Germany was infected with a similar virus for ≈3 years since 2014 (the first strain detected during the prolonged shedding, Leipzig07788a, is shown in the Figure) (*8*). Detection of these viruses as early as 1998 (Appendix 1 Table) suggests long-term circulation of these nontypeable strains. Their long-term geographically limited detection could result from multiple factors including, but not limited, to low transmissibility, restrictions on the mutability of the virus, host-related susceptibility (*9*), or underdetection because of limited norovirus surveillance or nucleotide mismatches in the PCR primers and probes for detection. In that regard, we found a substitution (C5047T) on a common probes binding site used for quantitative PCR detection in 6 nontypeable strains (Appendix 2). Further studies would provide insight into the apparent limited geographic distribution of these viruses.

Although only a few genotypes (e.g., GII.4, GII.3, GII.2, GII.6) are most prevalent among humans, those with limited circulation can emerge, causing large outbreaks (e.g., GII.17) (*10*,*11*). Monitoring of minor genotypes is helpful for preparing for the emergence of novel viruses and possible future outbreaks. Information about the circulation of each of the norovirus genotypes will provide clues to the natural history of norovirus disease and guide the development of specific treatment and prevention plans. 

Appendix 1 Supplemental data for study of recombinant nontypeable genotype GII human noroviruses in the Americas.

Appendix 2 Table showing mismatches of common quantitative PCR primer sequences.
