# Supplementary material for: Recombinant Nontypeable Genotype II Human Noroviruses in the Americas
Source: Emerg Infect Dis. 2020 Jan;26(1):157–9. doi: 10.3201/eid2601.190626 (PMC6924879; doi:10.3201/eid2601.190626)
Supplement: Appendix 1 — Supplemental data for study of recombinant nontypeable genotype GII human noroviruses in the Americas. [file 19-0626-Techapp-s1.pdf]

Article DOI: https://doi.org/10.3201/eid2601.190626

Recombinant Nontypeable Genotype II Human Noroviruses in the Americas

Appendix 1

Appendix 1 Table. Demographic and clinical information of the seven cases

Sample	Age	Symptom	Location	Date of collection	Note	Accession number
Arg1382	2 y.o.	AGE*	Los Polvorines, Buenos Aires, Argentina	4/3/1998	Outpatient	MK733201
Arg15465	11 m.o.	AGE	Buenos Aires City, Argentina	7/4/2017	Outpatient	MK733202
Arg15482	3 y.o.	AGE	Buenos Aires City, Argentina	8/1/2017	Outpatient	MK733203
Arg15559	1 y.o.	AGE	Buenos Aires City, Argentina	9/26/2017	Outpatient	MK733204
Arg15813	19 y.o.	AGE	Buenos Aires City, Argentina	1/9/2018	Intestine transplant in 2008 (Inmmunocompromised)	MK733205
PNV024019	1 y.o.	AGE	Lima, Peru	2/12/2010	Cohort (Saito M. et al. 2014)	MK733206
PNV027026	1 y.o.	AGE	Lima, Peru	6/11/2010	Cohort (Saito M. et al. 2014)	ML733207

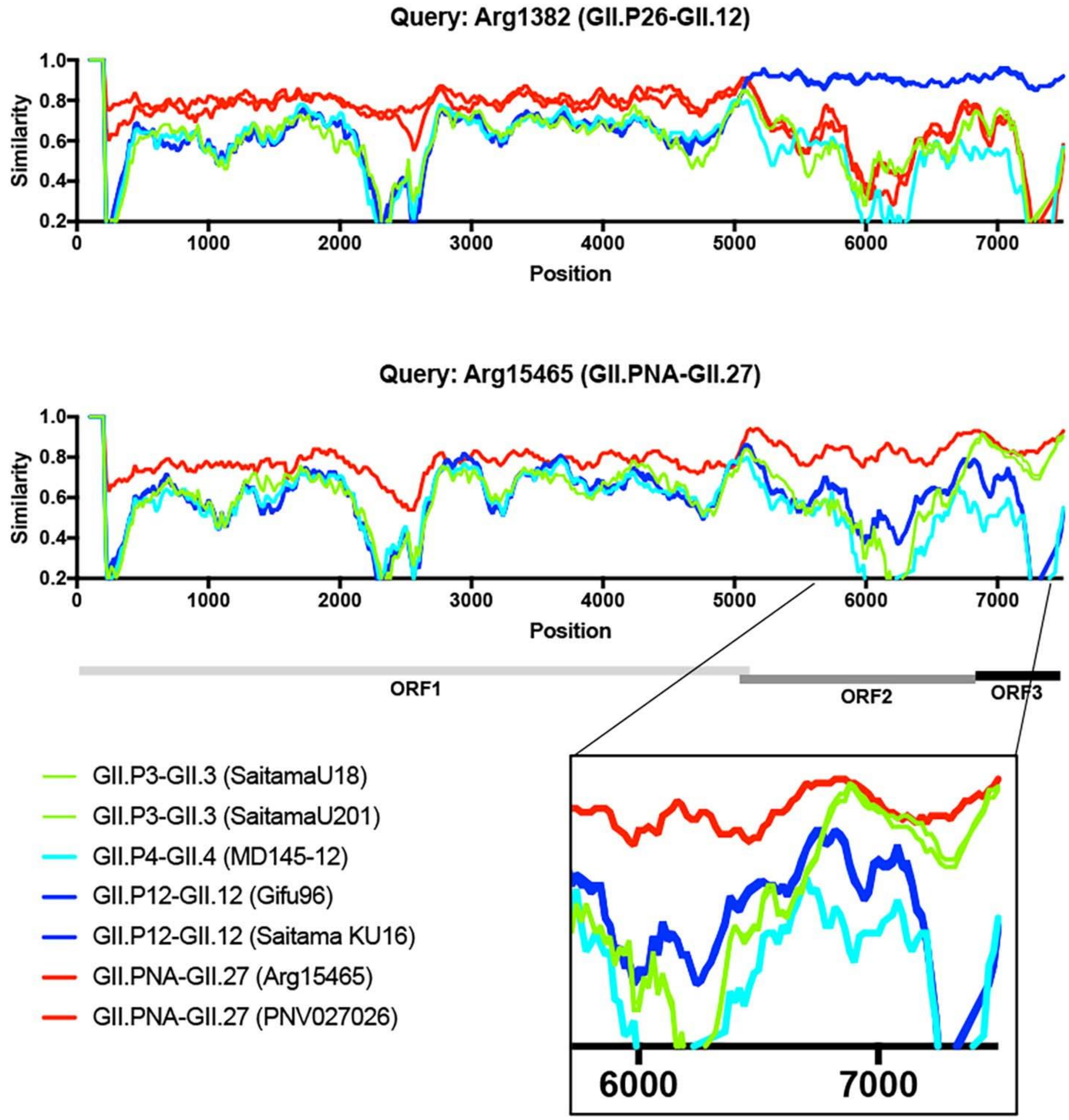

Appendix 1 Figure 1. Simplot analyses of the Arg1382 and the ARG15465 against norovirus GII.3, GII.4, GII.12, and GII.27 complete genome sequences. The x-axis indicates the nucleotide position of the genome and the y-axis indicates the nucleotide similarity against the query sequences. Lines were colored by capsid genotypes. Inset shows the details of genomic similarity and potential recombination of the VP2-encoding region.

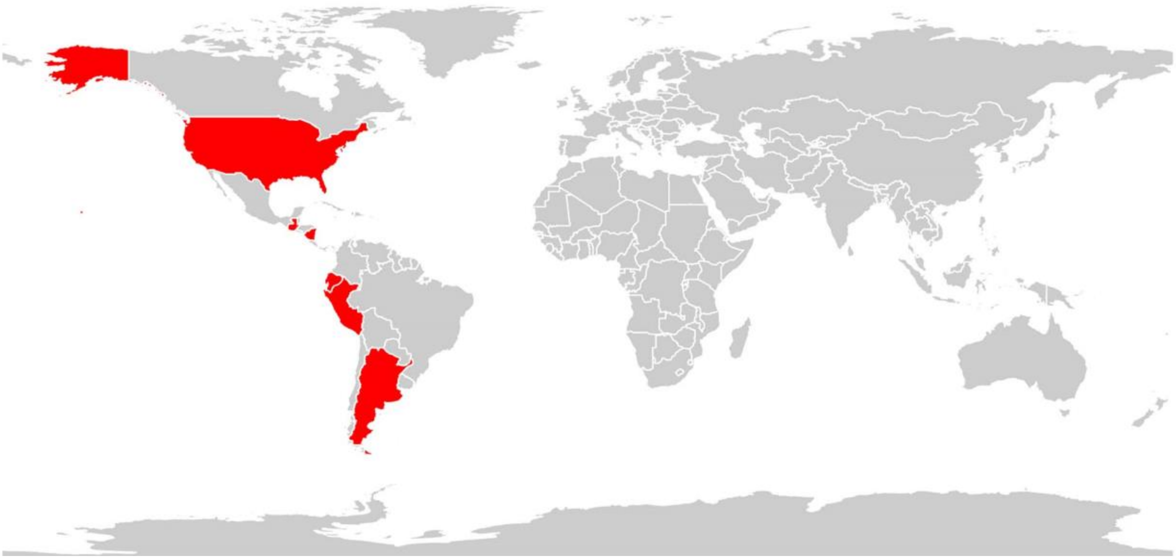

Appendix 1 Figure 2. Geographic distribution of nontypeable norovirus GII strains, indicating their local transmission in the Americas. Red indicates countries where the nontypeabe GII strains were detected.
